# 1-[(*E*)-(2-Phenoxy­anilino)methyl­ene]naphthalen-2(1*H*)-one

**DOI:** 10.1107/S1600536810013851

**Published:** 2010-04-21

**Authors:** Ersin Temel, Erbil Ağar, Orhan Büyükgüngör

**Affiliations:** a Department of Physics, Faculty of Arts and Sciences, Ondokuz Mayıs University, Kurupelit, TR-55139 Samsun, Turkey; b Department of Chemistry, Faculty of Arts and Sciences, Ondokuz Mayıs University, Kurupelit, TR-55139 Samsun, Turkey

## Abstract

The mol­ecule of the title compound, C_23_H_17_NO_2_, a Schiff base derived from 2-hydr­oxy-1-naphthaldehyde, crystallizes in the keto–amine tautomeric form. The dihedral angle between the aniline and hydroxy­benzene rings is 77.41 (17)°, whereas the planes of the naphthaldehyde and fused aniline benzene rings are nearly coplanar, making a dihedral angle of 8.29 (15)°. Intra­molecular N—H⋯O hydrogen bonding, a characteristic hydrogen bond for Schiff bases, helps to stabilize the mol­ecular structure. Weak inter­molecular C—H⋯π inter­actions are present in the crystal structure.

## Related literature

For Schiff bases, see: Caligaris *et al.* (1972 ▶); Caligaris & Randaccio *et al.* (1987 ▶); Salman *et al.* (1990 ▶); Popović *et al.* (2001 ▶); Garnovskii *et al.* (1993 ▶); Pyrz *et al.* (1985 ▶); Hadjoudis *et al.* (1987 ▶). For the HOMA (harmonic oscillator model of aromaticity) index, see: Krygowski *et al.* (1993 ▶). For similar structures, see: Özek *et al.* (2004 ▶); Takano *et al.* (2009 ▶).
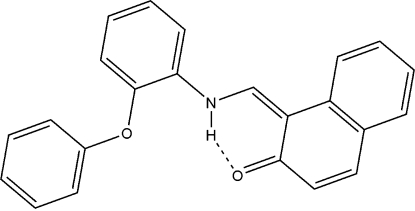


## Experimental

### 

#### Crystal data

C_23_H_17_NO_2_
*M* *_r_* = 339.38
Monoclinic, 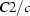

*a* = 14.6428 (11) Å
*b* = 5.6297 (3) Å
*c* = 42.602 (3) Å
β = 101.175 (6)°
*V* = 3445.3 (4) Å^3^
*Z* = 8
Mo *K*α radiation
μ = 0.08 mm^−1^
*T* = 296 K
0.40 × 0.34 × 0.23 mm

#### Data collection

Stoe IPDSII diffractometer
Absorption correction: integration (*X-RED*; Stoe & Cie, 2002 ▶) *T*_min_ = 0.960, *T*_max_ = 0.996
11832 measured reflections
2565 independent reflections
1522 reflections with *I* > 2σ(*I*)
*R*_int_ = 0.212
θ_max_ = 23.6°

#### Refinement

*R*[*F* ^2^ > 2σ(*F* ^2^)] = 0.115
*wR*(*F* ^2^) = 0.331
*S* = 1.06
2565 reflections
236 parameters
H-atom parameters constrained
Δρ_max_ = 0.30 e Å^−3^
Δρ_min_ = −0.39 e Å^−3^

### 

Data collection: *X-AREA* (Stoe & Cie, 2002 ▶); cell refinement: *X-AREA*; data reduction: *X-RED32* (Stoe & Cie, 2002 ▶); program(s) used to solve structure: *SHELXS97* (Sheldrick, 2008 ▶); program(s) used to refine structure: *SHELXL97* (Sheldrick, 2008 ▶); molecular graphics: *ORTEP-3 for Windows* (Farrugia, 1997 ▶); software used to prepare material for publication: *WinGX* (Farrugia, 1999 ▶).

## Supplementary Material

Crystal structure: contains datablocks I, global. DOI: 10.1107/S1600536810013851/si2256sup1.cif

Structure factors: contains datablocks I. DOI: 10.1107/S1600536810013851/si2256Isup2.hkl

Additional supplementary materials:  crystallographic information; 3D view; checkCIF report

## Figures and Tables

**Table 1:** Hydrogen-bond geometry (Å, °) *Cg*1 and *Cg*2 are the centroids of the C1–C6 and C18–C23 rings, respectively.

*D*—H⋯*A*	*D*—H	H⋯*A*	*D*⋯*A*	*D*—H⋯*A*
N1—H1⋯O1	0.86	1.87	2.561 (6)	136
C11—H11⋯*Cg*1^i^	0.93	2.87	3.664 (7)	144
C19—H19⋯*Cg*2^ii^	0.93	2.90	3.674 (7)	142

Symmetry codes: (i) 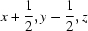; (ii) 
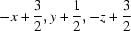
.

**Fig. 1.**
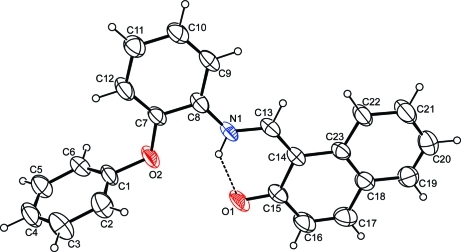
The molecular structure of (I), showing the atom-numbering scheme. Displacement ellipsoids are drawn at the 40% probability.

**Fig. 2.**
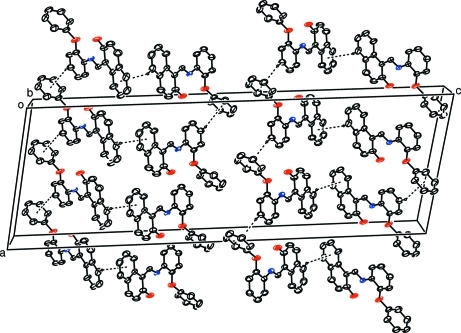
The packing of (I), showing weak C—H···π ring interactions with dashed lines.

## supplementary crystallographic information

### Comment

2-Hydroxy-Schiff bases are formed by reactions of salicylaldehyde and 2-hydroxy-1-naphthaldehyde with various amines (Caligaris *et al.*, 1972). In this study, a Schiff base derivatived from 2-hydroxy-1-naphthaldehyde was examined. In contrast to salicylaldimine derivatives, the Schiff bases of 2-hydroxy-1-naphthaldehyde have been rarely investigated (Salman *et al.*, 1990; Popović *et al.*, 2001). Schiff base ligands play a vital role in coordination chemistry due to their metal binding ability (Garnovskii *et al.*, 1993). In addition, Schiff bases and their metal complexes have wide applications in biological systems (Pyrz *et al.*, 1985).

The structure of *o*-hydroxy aromatic Schiff base has drawn attention due to their keto-enol tautomerism in recent years (Hadjoudis *et al.*, 1987). As being characteristic feature of Schiff bases there are two alternative intra-molecular hydrogen bonds depending on the type of tautomer. The structure with intra-molecular N—H···O hydrogen bond is called keto tautomer. The enol tautomer is, on the other hand, a structure involving O—H···N type hydrogen bond (Caligaris & Randaccio *et al.*, 1987). Details of hydrogen bond geometry are given in Table 1.

The proton transfer responsible for the tautomerization requires a small amount of energy which can be obtained by temperature change or light (Caligaris & Randaccio *et al.*, 1987). The proton transfer reaction causes the bond distances to deviate from the ideal value 1.338 Å, which leads to a decrease in aromaticity of the ring. In order to investigate deformation in π-electron delocalization of aromatic rings, HOMA (Harmonic Oscillator Model of Aromaticity) index is a useful tool. The HOMA index is equal to unity for purely aromatic systems and zero for non-aromatic systems (Krygowski *et al.*, 1993). The HOMA index of the naphthalene ring of (I) was calculated as 0.670, which is fairly less than the HOMA index of aromatic naphthalene. It can be inferred that π-electron delocalization of the ring involving proton transfer is considerably deformed.

The molecule of (I) is generated by connecting 2-phenoxyaniline and naphthaldehyde units through a nitrogen bridge (Figure 1). Rings A(C1—C6), B(C7—C12) and C(C14—C23) are planar and the dihedral angles between them are A/B=77.41 (17)°, A/C=79.24 (15)°, B/C=8.29 (15)°. The hydrogen atom in the title compound (I) is located on nitrogen atom, thus the keto-amine tautomer is favored over the phenol-imine form. The presence of keto form can be also confirmed by N1—C13 and C15—O1 bond lengths. The C15—O1 bond length of 1.276 (7)Å indicates double-bond character while the N1—C13 bond length of 1.315 (6)Å indicates single-bond character. Similar results are also reported in the literature (Özek *et al.*, 2004; Takano *et al.*, 2009]. Intermolecular weak C—H···π ring interactions are also present in the crystal structure (Figure 2). Details of C—H···π contacts are given in Table 1.

### Experimental

(*E*)-1-((2-phenoxyphenylamino)methyl)naphthalen-2-ol was prepared by refluxing a mixture of a solution containing 2-hydroxy-1-naphthaldehyde (17.2 mg, 0.1 mmol) in ethanol (30 ml) and a solution containing 2-phenoxyaniline (18.5 mg, 0.1 mmol) in ethanol (20 ml). The reaction mixture was stirred for 1 h. under reflux. Single crystals of the title compound for x-ray analysis were obtained by slow evaporation of an ethanol solution (Yield 68%; m.p. 411-413 K ).

### Refinement

H atoms attached to carbon atoms were placed in calculated positions with U_iso_(H) = 1.2U_eq_(C). The coordinates of the amine hydrogen obtained from a difference map and refined isotropically with N—H = 0.86Å constrain.

### Crystal data

**Table:**

C_23_H_17_NO_2_	*F*(000) = 1424
*M**_r_* = 339.38	*D*_x_ = 1.309 Mg m^−^^3^
Monoclinic, *C*2/*c*	Melting point = 411–413 K
Hall symbol: -C 2yc	Mo *K*α radiation, λ = 0.71073 Å
*a* = 14.6428 (11) Å	Cell parameters from 2565 reflections
*b* = 5.6297 (3) Å	θ = 2.8–24.1°
*c* = 42.602 (3) Å	µ = 0.08 mm^−^^1^
β = 101.175 (6)°	*T* = 296 K
*V* = 3445.3 (4) Å^3^	Long prismatic rod, yellow
*Z* = 8	0.40 × 0.34 × 0.23 mm

### Data collection

**Table:**

Stoe IPDSII diffractometer	2565 independent reflections
Radiation source: fine-focus sealed tube	1522 reflections with *I* > 2σ(*I*)
plane graphite	*R*_int_ = 0.212
Detector resolution: 6.67 pixels mm^-1^	θ_max_ = 23.6°, θ_min_ = 2.8°
rotation scans	*h* = −16→16
Absorption correction: integration (*X-RED*; Stoe & Cie, 2002)	*k* = −6→6
*T*_min_ = 0.960, *T*_max_ = 0.996	*l* = −47→47
11832 measured reflections	

### Refinement

**Table:**

Refinement on *F*^2^	Secondary atom site location: difference Fourier map
Least-squares matrix: full	Hydrogen site location: inferred from neighbouring sites
*R*[*F*^2^ > 2σ(*F*^2^)] = 0.115	H-atom parameters constrained
*wR*(*F*^2^) = 0.331	*w* = 1/[σ^2^(*F*_o_^2^) + (0.1935*P*)^2^] where *P* = (*F*_o_^2^ + 2*F*_c_^2^)/3
*S* = 1.06	(Δ/σ)_max_ = 0.001
2565 reflections	Δρ_max_ = 0.30 e Å^−^^3^
236 parameters	Δρ_min_ = −0.39 e Å^−^^3^
0 restraints	Extinction correction: *SHELXL97* (Sheldrick, 2008), Fc^*^=kFc[1+0.001xFc^2^λ^3^/sin(2θ)]^-1/4^
Primary atom site location: structure-invariant direct methods	Extinction coefficient: 0.0045 (17)

### Special details

**Table:**

Experimental. All esds (except the esd in the dihedral angle between two l.s. planes) are estimated using the full covariance matrix. The cell esds are taken into account individually in the estimation of esds in distances, angles and torsion angles; correlations between esds in cell parameters are only used when they are defined by crystal symmetry. An approximate (isotropic) treatment of cell esds is used for estimating esds involving l.s. planes.
Geometry. All esds (except the esd in the dihedral angle between two l.s. planes) are estimated using the full covariance matrix. The cell esds are taken into account individually in the estimation of esds in distances, angles and torsion angles; correlations between esds in cell parameters are only used when they are defined by crystal symmetry. An approximate (isotropic) treatment of cell esds is used for estimating esds involving l.s. planes.
Refinement. Refinement of *F*^2^ against ALL reflections. The weighted *R*-factor wR and goodness of fit *S* are based on *F*^2^, conventional *R*-factors *R* are based on *F*, with *F* set to zero for negative *F*^2^. The threshold expression of *F*^2^ > σ(*F*^2^) is used only for calculating *R*-factors(gt) etc. and is not relevant to the choice of reflections for refinement. *R*-factors based on *F*^2^ are statistically about twice as large as those based on *F*, and *R*- factors based on ALL data will be even larger.

### Fractional atomic coordinates and isotropic or equivalent isotropic displacement parameters (Å^2^)

**Table:**

	*x*	*y*	*z*	*U*_iso_*/*U*_eq_	
C1	0.4850 (4)	−0.3268 (11)	0.56310 (14)	0.0576 (16)	
C2	0.4649 (5)	−0.1907 (13)	0.53616 (18)	0.0748 (19)	
H2	0.4969	−0.0499	0.5345	0.090*	
C3	0.3949 (5)	−0.2683 (15)	0.51107 (16)	0.082 (2)	
H3	0.3797	−0.1800	0.4923	0.099*	
C4	0.3489 (4)	−0.4759 (13)	0.51444 (17)	0.075 (2)	
H4	0.3040	−0.5314	0.4976	0.090*	
C5	0.3683 (4)	−0.6017 (12)	0.54216 (17)	0.0704 (19)	
H5	0.3345	−0.7383	0.5445	0.084*	
C6	0.4377 (4)	−0.5276 (11)	0.56682 (15)	0.0641 (18)	
H6	0.4517	−0.6144	0.5857	0.077*	
C7	0.6418 (3)	−0.3294 (10)	0.59332 (14)	0.0513 (14)	
C8	0.7060 (3)	−0.2112 (10)	0.61624 (13)	0.0482 (14)	
C9	0.7982 (4)	−0.2933 (11)	0.62192 (15)	0.0581 (16)	
H9	0.8429	−0.2158	0.6369	0.070*	
C10	0.8231 (4)	−0.4821 (11)	0.60609 (16)	0.0622 (17)	
H10	0.8845	−0.5343	0.6106	0.075*	
C11	0.7588 (4)	−0.5997 (11)	0.58329 (15)	0.0627 (17)	
H11	0.7767	−0.7284	0.5722	0.075*	
C12	0.6665 (4)	−0.5216 (12)	0.57725 (15)	0.0639 (18)	
H12	0.6220	−0.6002	0.5623	0.077*	
C13	0.7229 (4)	0.1281 (10)	0.65264 (13)	0.0503 (14)	
H13	0.7872	0.1090	0.6571	0.060*	
C14	0.6860 (4)	0.3109 (9)	0.66784 (13)	0.0487 (14)	
C15	0.5865 (4)	0.3495 (10)	0.66058 (15)	0.0571 (16)	
C16	0.5513 (4)	0.5463 (12)	0.67467 (18)	0.0716 (19)	
H16	0.4876	0.5747	0.6699	0.086*	
C17	0.6065 (4)	0.6971 (12)	0.69492 (18)	0.0704 (19)	
H17	0.5797	0.8235	0.7039	0.084*	
C18	0.7048 (4)	0.6642 (10)	0.70262 (14)	0.0548 (15)	
C19	0.7619 (5)	0.8288 (11)	0.72230 (14)	0.0647 (17)	
H19	0.7347	0.9566	0.7308	0.078*	
C20	0.8549 (5)	0.8045 (12)	0.72899 (16)	0.0730 (19)	
H20	0.8916	0.9137	0.7422	0.088*	
C21	0.8956 (5)	0.6163 (13)	0.71618 (17)	0.078 (2)	
H21	0.9599	0.5976	0.7213	0.094*	
C22	0.8435 (4)	0.4567 (12)	0.69608 (16)	0.0691 (19)	
H22	0.8728	0.3352	0.6870	0.083*	
C23	0.7448 (4)	0.4752 (9)	0.68896 (12)	0.0481 (14)	
N1	0.6738 (3)	−0.0213 (8)	0.63234 (10)	0.0493 (12)	
H1	0.6146	0.0005	0.6285	0.059*	
O1	0.5318 (2)	0.2158 (8)	0.64128 (11)	0.0720 (14)	
O2	0.5522 (2)	−0.2386 (8)	0.58887 (11)	0.0755 (15)	

### Atomic displacement parameters (Å^2^)

**Table:**

	*U*^11^	*U*^22^	*U*^33^	*U*^12^	*U*^13^	*U*^23^
C1	0.031 (3)	0.080 (4)	0.055 (4)	0.002 (3)	−0.008 (3)	−0.025 (3)
C2	0.060 (4)	0.084 (4)	0.075 (5)	−0.016 (3)	−0.001 (3)	−0.005 (4)
C3	0.072 (5)	0.116 (6)	0.050 (4)	−0.011 (4)	−0.010 (3)	0.007 (4)
C4	0.044 (3)	0.098 (5)	0.072 (5)	−0.011 (3)	−0.014 (3)	−0.016 (4)
C5	0.045 (3)	0.084 (4)	0.075 (5)	−0.010 (3)	−0.005 (3)	−0.007 (4)
C6	0.049 (3)	0.074 (4)	0.060 (4)	0.001 (3)	−0.013 (3)	−0.002 (3)
C7	0.028 (3)	0.066 (3)	0.055 (3)	0.000 (2)	−0.003 (2)	−0.011 (3)
C8	0.033 (3)	0.068 (3)	0.042 (3)	−0.005 (2)	0.003 (2)	−0.006 (3)
C9	0.031 (3)	0.075 (4)	0.062 (4)	−0.010 (3)	−0.007 (3)	0.001 (3)
C10	0.032 (3)	0.077 (4)	0.074 (4)	−0.003 (3)	0.003 (3)	−0.008 (3)
C11	0.043 (3)	0.080 (4)	0.064 (4)	0.001 (3)	0.008 (3)	−0.014 (3)
C12	0.033 (3)	0.093 (4)	0.061 (4)	−0.007 (3)	−0.002 (3)	−0.022 (3)
C13	0.033 (3)	0.071 (3)	0.043 (3)	0.000 (3)	−0.003 (2)	−0.001 (3)
C14	0.034 (3)	0.062 (3)	0.046 (3)	−0.003 (2)	−0.003 (2)	0.001 (3)
C15	0.029 (3)	0.069 (4)	0.069 (4)	−0.003 (3)	0.000 (3)	−0.001 (3)
C16	0.034 (3)	0.090 (5)	0.088 (5)	0.006 (3)	0.004 (3)	−0.012 (4)
C17	0.048 (3)	0.080 (4)	0.083 (5)	0.014 (3)	0.012 (3)	−0.004 (4)
C18	0.049 (3)	0.061 (3)	0.050 (3)	0.004 (3)	0.000 (3)	0.000 (3)
C19	0.073 (4)	0.065 (4)	0.050 (3)	0.008 (3)	−0.001 (3)	−0.008 (3)
C20	0.070 (4)	0.082 (4)	0.060 (4)	−0.009 (4)	−0.005 (3)	−0.013 (3)
C21	0.045 (3)	0.106 (5)	0.075 (5)	−0.004 (3)	−0.007 (3)	−0.021 (4)
C22	0.038 (3)	0.093 (4)	0.070 (4)	0.003 (3)	−0.007 (3)	−0.034 (3)
C23	0.041 (3)	0.058 (3)	0.040 (3)	0.003 (2)	−0.004 (2)	0.002 (2)
N1	0.027 (2)	0.071 (3)	0.045 (3)	−0.006 (2)	−0.0059 (19)	−0.006 (2)
O1	0.0240 (19)	0.097 (3)	0.087 (3)	−0.0049 (19)	−0.009 (2)	−0.018 (3)
O2	0.028 (2)	0.106 (3)	0.083 (3)	0.004 (2)	−0.013 (2)	−0.047 (3)

### Geometric parameters (Å, °)

**Table:**

C1—C6	1.351 (9)	C12—H12	0.9300
C1—C2	1.364 (9)	C13—N1	1.315 (6)
C1—O2	1.414 (6)	C13—C14	1.381 (8)
C2—C3	1.400 (9)	C13—H13	0.9300
C2—H2	0.9300	C14—C15	1.447 (7)
C3—C4	1.370 (10)	C14—C23	1.451 (7)
C3—H3	0.9300	C15—O1	1.276 (7)
C4—C5	1.359 (10)	C15—C16	1.404 (9)
C4—H4	0.9300	C16—C17	1.359 (9)
C5—C6	1.377 (8)	C16—H16	0.9300
C5—H5	0.9300	C17—C18	1.425 (8)
C6—H6	0.9300	C17—H17	0.9300
C7—C12	1.366 (8)	C18—C23	1.395 (8)
C7—O2	1.387 (6)	C18—C19	1.411 (8)
C7—C8	1.387 (7)	C19—C20	1.344 (10)
C8—N1	1.400 (7)	C19—H19	0.9300
C8—C9	1.403 (8)	C20—C21	1.379 (10)
C9—C10	1.347 (9)	C20—H20	0.9300
C9—H9	0.9300	C21—C22	1.368 (8)
C10—C11	1.383 (8)	C21—H21	0.9300
C10—H10	0.9300	C22—C23	1.422 (8)
C11—C12	1.397 (8)	C22—H22	0.9300
C11—H11	0.9300	N1—H1	0.8600
			
C6—C1—C2	122.4 (5)	N1—C13—H13	117.6
C6—C1—O2	119.9 (6)	C14—C13—H13	117.6
C2—C1—O2	117.4 (6)	C13—C14—C15	118.8 (5)
C1—C2—C3	118.3 (7)	C13—C14—C23	121.8 (5)
C1—C2—H2	120.9	C15—C14—C23	119.3 (5)
C3—C2—H2	120.9	O1—C15—C16	120.3 (5)
C4—C3—C2	119.2 (7)	O1—C15—C14	122.0 (5)
C4—C3—H3	120.4	C16—C15—C14	117.7 (5)
C2—C3—H3	120.4	C17—C16—C15	122.9 (5)
C5—C4—C3	120.8 (6)	C17—C16—H16	118.6
C5—C4—H4	119.6	C15—C16—H16	118.6
C3—C4—H4	119.6	C16—C17—C18	120.9 (6)
C4—C5—C6	120.3 (6)	C16—C17—H17	119.5
C4—C5—H5	119.9	C18—C17—H17	119.5
C6—C5—H5	119.9	C23—C18—C19	120.0 (5)
C1—C6—C5	118.9 (6)	C23—C18—C17	119.4 (5)
C1—C6—H6	120.5	C19—C18—C17	120.5 (6)
C5—C6—H6	120.5	C20—C19—C18	121.3 (6)
C12—C7—O2	124.0 (5)	C20—C19—H19	119.4
C12—C7—C8	121.5 (5)	C18—C19—H19	119.4
O2—C7—C8	114.4 (5)	C19—C20—C21	119.5 (6)
C7—C8—N1	117.7 (5)	C19—C20—H20	120.2
C7—C8—C9	117.5 (5)	C21—C20—H20	120.2
N1—C8—C9	124.8 (5)	C22—C21—C20	121.4 (6)
C10—C9—C8	121.3 (5)	C22—C21—H21	119.3
C10—C9—H9	119.4	C20—C21—H21	119.3
C8—C9—H9	119.4	C21—C22—C23	120.4 (6)
C9—C10—C11	121.0 (6)	C21—C22—H22	119.8
C9—C10—H10	119.5	C23—C22—H22	119.8
C11—C10—H10	119.5	C18—C23—C22	117.3 (5)
C10—C11—C12	118.8 (6)	C18—C23—C14	119.9 (5)
C10—C11—H11	120.6	C22—C23—C14	122.8 (5)
C12—C11—H11	120.6	C13—N1—C8	128.1 (4)
C7—C12—C11	119.9 (5)	C13—N1—H1	115.9
C7—C12—H12	120.1	C8—N1—H1	115.9
C11—C12—H12	120.1	C7—O2—C1	118.3 (4)
N1—C13—C14	124.8 (5)		
			
C6—C1—C2—C3	−2.5 (11)	C14—C15—C16—C17	−1.0 (11)
O2—C1—C2—C3	−176.5 (6)	C15—C16—C17—C18	1.0 (11)
C1—C2—C3—C4	0.3 (11)	C16—C17—C18—C23	0.4 (10)
C2—C3—C4—C5	2.4 (12)	C16—C17—C18—C19	176.6 (7)
C3—C4—C5—C6	−3.0 (11)	C23—C18—C19—C20	−1.8 (10)
C2—C1—C6—C5	1.9 (10)	C17—C18—C19—C20	−177.9 (7)
O2—C1—C6—C5	175.7 (6)	C18—C19—C20—C21	0.6 (11)
C4—C5—C6—C1	0.9 (10)	C19—C20—C21—C22	1.7 (12)
C12—C7—C8—N1	−177.8 (5)	C20—C21—C22—C23	−2.7 (12)
O2—C7—C8—N1	1.2 (8)	C19—C18—C23—C22	0.7 (9)
C12—C7—C8—C9	1.2 (9)	C17—C18—C23—C22	176.9 (6)
O2—C7—C8—C9	−179.8 (5)	C19—C18—C23—C14	−177.9 (5)
C7—C8—C9—C10	−1.0 (9)	C17—C18—C23—C14	−1.7 (9)
N1—C8—C9—C10	177.9 (6)	C21—C22—C23—C18	1.5 (10)
C8—C9—C10—C11	1.0 (10)	C21—C22—C23—C14	−180.0 (6)
C9—C10—C11—C12	−1.1 (10)	C13—C14—C23—C18	176.9 (5)
O2—C7—C12—C11	179.8 (6)	C15—C14—C23—C18	1.6 (8)
C8—C7—C12—C11	−1.4 (10)	C13—C14—C23—C22	−1.6 (9)
C10—C11—C12—C7	1.3 (10)	C15—C14—C23—C22	−176.9 (6)
N1—C13—C14—C15	−2.0 (9)	C14—C13—N1—C8	−179.5 (5)
N1—C13—C14—C23	−177.4 (5)	C7—C8—N1—C13	−173.3 (5)
C13—C14—C15—O1	2.0 (9)	C9—C8—N1—C13	7.7 (9)
C23—C14—C15—O1	177.4 (6)	C12—C7—O2—C1	−9.5 (10)
C13—C14—C15—C16	−175.8 (6)	C8—C7—O2—C1	171.6 (6)
C23—C14—C15—C16	−0.3 (9)	C6—C1—O2—C7	84.9 (8)
O1—C15—C16—C17	−178.8 (7)	C2—C1—O2—C7	−101.0 (7)

### Hydrogen-bond geometry (Å, °)

**Table:**

Cg1 and Cg2 are the centroids of the C1–C6 and C18–C23 rings, respectively.

**Table:**

*D*—H···*A*	*D*—H	H···*A*	*D*···*A*	*D*—H···*A*
N1—H1···O1	0.86	1.87	2.561 (6)	136
C11—H11···Cg1^i^	0.93	2.87	3.664 (7)	144
C19—H19···Cg2^ii^	0.93	2.90	3.674 (7)	142

Symmetry codes: (i) *x*+1/2, *y*−1/2, *z*; (ii) −*x*+3/2, *y*+1/2, −*z*+3/2.
